# Supplementary material for: A computational method for drug sensitivity prediction of cancer cell lines based on various molecular information
Source: PLoS One. 2021 Apr 29;16(4):e0250620. doi: 10.1371/journal.pone.0250620 (PMC8084246; doi:10.1371/journal.pone.0250620)
Supplement: S2 File — (PDF) [file pone.0250620.s002.pdf]

A Computational Method for Drug Sensitivity Prediction of Cancer Cell Lines Based on Various Molecular Information

Fatemeh Ahmadi Moughari, Changiz Eslahchi
Corresponding author: Changiz Eslahchi- Contact mail: ch-eslahchi@sbu.ac.ir

Supplementary Materials

Contents

1 The detailed formulae of manifold leaning in CDSML **3**

2 The detailed formulae for handling the response matrix with missing values **4**

3 The detailed formulae for single, or no similarity scenarios **5**
- 3.1 Single scenario 6
- 3.2 No similarity scenario 7

4 The performance of CDSML on CCLE dataset using different scenarios **8**

5 Comparison of Zhang et al. predictions with imputed labels **9**

6 Comparison of Zhang et al. predictions with CDSML predicted labels **9**
- 6.1 Comparison on missing pairs 9
- 6.2 Comparison on known pairs 10

7 Supplementary Figure 1 **10**

8 Supplementary Figure 2 **10**

1 The detailed formulae of manifold leaning in CDSML

The manifold learning tries to decompose $B_{m\times n}$ matrix into latent matrices $X_{m\times k}$ and $Y_{n\times k}$, such that $B \approx XY^T$. Therefore we aim to minimize the difference between B and $B \approx$. So the initial loss function is defined in Eq. 1.

$$Loss^{(initial)} = \frac{1}{2}\sum_{i,j}(B(i,j) - X(i)Y(j)^T)^2 \tag{1}$$

Moreover, it is not favorable for the latent matrices to have high norm because it may put the threat of high variance for the model. Therefore, we aim to minimize the norm of latent matrices. The improved version of loss function is defined in Eq. 2.

$$Loss^{(initial)} = \frac{1}{2}\sum_{i,j}(B(i,j) - X(i)Y(j)^T)^2 + \frac{\alpha}{2}\left(\sum_i ||X(i)||^2 + \sum_j ||Y(j)||^2\right) \tag{2}$$

Furthermore, it is desirable to learn the latent matrices such that it preserve the manifold properties of the samples. Clearly speaking, we aim to find the latent matrices such that for any pair of cell lines c_i and c_j, the distance of c_i and c_j can be estimated by the euclidean distance of their latent vectors. In other words, for c_i and c_j pairs that have high similarity, $||X(i) - X(j)||^2$ must not be a great value. The similar constraint should be satisfied for drugs. Therefore, the final loss function is defined in Eq. 3.

$$\begin{aligned} Loss = {} & \frac{1}{2}\sum_{i,j}(B(i,j) - X(i)Y(j)^T)^2 + \frac{\alpha}{2}\left(\sum_i ||X(i)||^2 + \sum_j ||Y(j)||^2\right) \\ & + \frac{\beta}{2}\left(\sum_{i,j} ||X(i) - X(j)||^2 SC(i,j) + \sum_{i,j} ||Y(i) - Y(j)||^2 SD(i,j)\right) \end{aligned} \tag{3}$$

where α and β are the regularization and similarity conservation coefficients.

Two latent matrices X and Y were updated using Newton's method to minimize the loss function iteratively. $X^{(0)}$ and $Y^{(0)}$ were initialized randomly and afterwards, $X^{(k)}$ and $Y^{(k)}$ were updated using the rules defined in Formulae 4 and 5, respectively.

$$X^{(k+1)} = X^{(k)} - \frac{\nabla_{X^{(k)}} Loss}{\nabla^2_{X^{(k)}} Loss} \tag{4}$$

$$Y^{(k+1)} = Y^{(k)} - \frac{\nabla_{Y^{(k)}} Loss}{\nabla^2_{Y^{(k)}} Loss} \tag{5}$$

Thus, we must compute $\nabla_{X^{(k)}} Loss$, $\nabla^2_{X^{(k)}} Loss$, $\nabla_{Y^{(k)}} Loss$, and $\nabla^2_{Y^{(k)}} Loss$ based on the following formulae:

$$\begin{aligned} \nabla_{X(i)^{(k)}} Loss = {} & \sum_{i,j}(X(i)Y(j)^T - B(i,j))Y(j) + \alpha * X(i) + \beta * \sum_j (X(i) - X(j))SC(i,j) \\ & - \beta * \sum_j (X(j) - X(i))SC(j,i) \end{aligned} \tag{6}$$

$$\nabla^2_{X(i)^{(k)}} Loss = \sum_{i,j} Y(j)^T Y(j) + \alpha I + \beta * \sum_j (SC(i,j) + SC(j,i)) I \tag{7}$$

$$\begin{aligned}\nabla_{Y(j)^{(k)}} Loss = \sum_{i,j} (X(i)Y(j)^T - B(i,j))X(i) + \alpha * Y(j) + \beta * \sum_i (Y(i) - Y(j))SD(i,j) \\ - \beta * \sum_i (Y(j) - Y(i))SD(j,i)\end{aligned} \tag{8}$$

$$\nabla^2_{Y(j)^{(k)}} Loss = \sum_{i,j} X(i)^T X(i) + \alpha I + \beta * \sum_i (SD(i,j) + SD(j,i)) I \tag{9}$$

Eventually, the latent vectors are updated using Eq. 10 and 11.

$$\begin{aligned}X(i)^{(k+1)} = &\left[\sum_{i,j} B(i,j)Y(j) + \beta * \sum_j (SC(i,j) + SC(j,i))X(i)^k\right] \\ &\left[\sum_{i,j} Y(j)^T Y(j) + \alpha I + \beta * \sum_j (SC(i,j) + SC(j,i)) I\right]^{-1}\end{aligned} \tag{10}$$

$$\begin{aligned}Y(j)^{(k+1)} = &\left[\sum_{i,j} B(i,j)X(i) + \beta * \sum_i (SD(i,j) + SD(j,i))Y(j)^k\right] \\ &\left[\sum_{i,j} X(i)^T X(i) + \alpha I + \beta * \sum_i (SD(i,j) + SD(j,i)) I\right]^{-1}\end{aligned} \tag{11}$$

These matrices were updated until $||X^{(k+1)} - X^{(k)}|| + ||Y^{(k+1)} - Y^{(k)}|| < 0.01$.

2 The detailed formulae for handling the response matrix with missing values

CDSML can handle the binary response matrix B without imputing missing values. To this aim, one should ignore computing the first term of loss function for missing pairs. Eq. 12 is capable of handling the response matrix containing missing values.

$$\begin{aligned}Loss^{(Missing)} = &\frac{1}{2} \sum_{i,j \notin Missing} (B(i,j) - X(i)Y(j)^T)^2 + \frac{\alpha}{2}\left(\sum_i ||X(i)||^2 + \sum_j ||Y(j)||^2\right) \\ &+ \frac{\beta}{2}\left(\sum_{i,j} ||X(i) - X(j)||^2 SC(i,j) + \sum_{i,j} ||Y(i) - Y(j)||^2 SD(i,j)\right)\end{aligned} \tag{12}$$

According to $Loss^{(Missing)}$, the $\nabla_{X^{(k)}} Loss^{(Missing)}$, $\nabla^2_{X^{(k)}} Loss^{(Missing)}$, $\nabla_{Y^{(k)}} Loss^{(Missing)}$, and $\nabla^2_{Y^{(k)}} Loss^{(Missing)}$ are computed based on the following formulae:

$$\begin{aligned}\nabla_{X(i)^{(k)}} Loss^{(Missing)} = &\sum_{i,j \notin Missing} (X(i)Y(j)^T - B(i,j))Y(j) + \alpha * X(i) \\ &+ \beta * \sum_j (X(i) - X(j))SC(i,j) - \beta * \sum_j (X(j) - X(i))SC(j,i)\end{aligned} \tag{13}$$

$$\nabla^2_{X(i)^{(k)}} Loss^{(Missing)} = \sum_{i,j \notin Missing} Y(j)^T Y(j) + \alpha I + \beta * \sum_j (SC(i,j) + SC(j,i))I \tag{14}$$

$$\begin{aligned}\nabla_{Y(j)^{(k)}} Loss^{(Missing)} = &\sum_{i,j \notin Missing} (X(i)Y(j)^T - B(i,j))X(i) + \alpha * Y(j) \\ &+ \beta * \sum_i (Y(i) - Y(j))SD(i,j) - \beta * \sum_j (Y(j) - Y(i))SD(j,i)\end{aligned} \tag{15}$$

$$\nabla^2_{Y(j)^{(k)}} Loss^{(Missing)} = \sum_{i,j \notin Missing} X(i)Y(j)^T + \alpha I + \beta * \sum_i (SD(i,j) + SD(j,i))I \tag{16}$$

Eventually, the latent vectors are updated using Eq. 17 and 18.

$$\begin{aligned}X(i)^{(k+1)} = &\left[\sum_{i,j \notin Missing} B(i,j)Y(j) + \beta * \sum_j (SC(i,j) + SC(j,i))X(i)^k\right] \\ &\left[\sum_{i,j \notin Missing} Y(j)^T Y(j) + \alpha I + \beta * \sum_j (SC(i,j) + SC(j,i))I\right]^{-1}\end{aligned} \tag{17}$$

$$\begin{aligned}Y(j)^{(k+1)} = &\left[\sum_{i,j \notin Missing} B(i,j)X(i) + \beta * \sum_i (SD(i,j) + SD(j,i))Y(j)^k\right] \\ &\left[\sum_{i,j \notin Missing} X(i)^T X(i) + \alpha I + \beta * \sum_i (SD(i,j) + SD(j,i))I\right]^{-1}\end{aligned} \tag{18}$$

3 The detailed formulae for single, or no similarity scenarios

The CDSML performance can be evaluated in three different scenarios: double similarity, single similarity, and no similarity. In case of using double similarity the formulae described in Section 1 are used. For single ans no similarity scenarios we used the formulae described in the following subsections.

3.1 Single scenario

If only SC is ignored, the loss function will be changed to the Eq. 19.

$$Loss^{(SD)} = \frac{1}{2} \sum_{i,j \notin Missing} (B(i,j) - X(i)Y(j)^T)^2 + \frac{\alpha}{2} \left(\sum_i ||X(i)||^2 + \sum_j ||Y(j)||^2 \right) + \frac{\beta}{2} \left(\sum_{i,j} ||Y(i) - Y(j)||^2 SD(i,j) \right) \tag{19}$$

Therefore, $\nabla_{X^{(k)}} Loss^{(SD)}$, $\nabla^2_{X^{(k)}} Loss^{(SD)}$, $\nabla_{Y^{(k)}} Loss^{(SD)}$, and $\nabla^2_{Y^{(k)}} Loss^{(SD)}$ are computed based on the following formulae:

$$\nabla_{X(i)^{(k)}} Loss^{(SD)} = \sum_{i,j \notin Missing} (X(i)Y(j)^T - B(i,j))Y(j) + \alpha * X(i) \tag{20}$$

$$\nabla^2_{X(i)^{(k)}} Loss^{(SD)} = \sum_{i,j \notin Missing} Y(j)^T Y(j) + \alpha I \tag{21}$$

$$\nabla_{Y(j)^{(k)}} Los^{(SD)}s = \sum_{i,j \notin Missing} (X(i)Y(j)^T - B(i,j))X(i) + \alpha * Y(j) + \beta * \sum_i (Y(i) - Y(j))SD(i,j) - \beta * \sum_i (Y(j) - Y(i))SD(j,i) \tag{22}$$

$$\nabla^2_{Y(j)^{(k)}} Loss^{(SD)} = \sum_{i,j \notin Missing} X(i)^T X(i) + \alpha I + \beta * \sum_i (SD(i,j) + SD(j,i))I \tag{23}$$

Eventually, the latent vectors are updated using Eq. 24 and 25.

$$X(i)^{(k+1)} = \left[\sum_{i,j \notin Missing} B(i,j)Y(j) \right] \left[\sum_{i,j \notin Missing} Y(j)^T Y(j) + \alpha I \right]^{-1} \tag{24}$$

$$Y(j)^{(k+1)} = \left[\sum_{i,j \notin Missing} B(i,j)X(i) + \beta * \sum_i (SD(i,j) + SD(j,i))Y(j)^k \right] \left[\sum_{i,j \notin Missing} X(i)^T X(i) + \alpha I + \beta * \sum_i (SD(i,j) + SD(j,i))I \right]^{-1} \tag{25}$$

If only SD is ignored, the loss function will be changed to the Eq. 26.

$$Loss^{(SC)} = \frac{1}{2} \sum_{i,j \notin Missing} (B(i,j) - X(i)Y(j)^T)^2 + \frac{\alpha}{2} \left(\sum_i ||X(i)||^2 + \sum_j ||Y(j)||^2 \right) + \frac{\beta}{2} \left(\sum_{i,j} ||X(i) - X(j)||^2 SC(i,j) \right) \tag{26}$$

Thus, we must compute $\nabla_{X^{(k)}} Loss^{(SC)}$, $\nabla^2_{X^{(k)}} Loss^{(SC)}$, $\nabla_{Y^{(k)}} Loss^{(SC)}$, and $\nabla^2_{Y^{(k)}} Loss^{(SC)}$ based on the following formulae:

$$\begin{aligned}\nabla_{X(i)^{(k)}} Loss^{(SC)} = &\sum_{i,j \notin Missing} (X(i)Y(j)^T - B(i,j))Y(j) + \alpha * X(i) + \beta * \sum_j (X(i) - X(j))SC(i,j) \\ &- \beta * \sum_j (X(j) - X(i))SC(j,i)\end{aligned} \tag{27}$$

$$\nabla^2_{X(i)^{(k)}} Loss^{(SC)} = \sum_{i,j \notin Missing} Y(j)^T Y(j) + \alpha I + \beta * \sum_j (SC(i,j) + SC(j,i))I \tag{28}$$

$$\nabla_{Y(j)^{(k)}} Loss^{(SC)} = \sum_{i,j \notin Missing} (X(i)Y(j)^T - B(i,j))X(i) + \alpha * Y(j) \tag{29}$$

$$\nabla^2_{Y(j)^{(k)}} Loss^{(SC)} = \sum_{i,j \notin Missing} X(i)^T X(i) + \alpha I \tag{30}$$

Eventually, the latent vectors are updated using Eq. 31 and 32.

$$\begin{aligned}X(i)^{(k+1)} = &\left[\sum_{i,j \notin Missing} B(i,j)Y(j) + \beta * \sum_j (SC(i,j) + SC(j,i))X(i)^k\right] \\ &\left[\sum_{i,j \notin Missing} Y(j)^T Y(j) + \alpha I + \beta * \sum_j (SC(i,j) + SC(j,i))I\right]^{-1}\end{aligned} \tag{31}$$

$$Y(j)^{(k+1)} = \left[\sum_{i,j} B(i,j \notin Missing)X(i)\right]\left[\sum_{i,j \notin Missing} X(i)^T X(i) + \alpha I\right]^{-1} \tag{32}$$

3.2 No similarity scenario

If both SC, SD are ignored, the loss function will be changed to the Eq. 33.

$$Loss^{(No\ sim)} = \frac{1}{2}\sum_{i,j \notin Missing} (B(i,j) - X(i)Y(j)^T)^2 + \frac{\alpha}{2}\left(\sum_i ||X(i)||^2 + \sum_j ||Y(j)||^2\right) \tag{33}$$

Thus, we must compute $\nabla_{X^{(k)}} Loss^{(No\ sim)}$, $\nabla^2_{X^{(k)}} Loss^{(No\ sim)}$, $\nabla_{Y^{(k)}} Loss^{(No\ sim)}$, and $\nabla^2_{Y^{(k)}} Loss^{(No\ sim)}$ based on the following formulae:

$$\nabla_{X(i)^{(k)}} Loss^{(No\ sim)} = \sum_{i,j \notin Missing} (X(i)Y(j)^T - B(i,j))Y(j) + \alpha * X(i) \tag{34}$$

$$\nabla^2_{X(i)^{(k)}} Loss^{(No\ sim)} = \sum_{i,j \notin Missing} Y(j)^T Y(j) + \alpha I \tag{35}$$

$$\nabla_{Y(j)^{(k)}} Loss^{(No\ sim)} = \sum_{i,j \notin Missing} (X(i)Y(j)^T - B(i,j))X(i) + \alpha * Y(j) \tag{36}$$

$$\nabla^2_{Y(j)^{(k)}} Loss^{(No\ sim)} = \sum_{i,j \notin Missing} X(i)^T X(i) + \alpha I \tag{37}$$

Eventually, the latent vectors are updated using Eq. 38 and 39.

$$X(i)^{(k+1)} = \left[\sum_{i,j \notin Missing} B(i,j)Y(j) \right] \left[\sum_{i,j \notin Missing} Y(j)^T Y(j) + \alpha I \right]^{-1} \tag{38}$$

$$Y(j)^{(k+1)} = \left[\sum_{i,j \notin Missing} B(i,j)X(i) \right] \left[\sum_{i,j \notin Missing} X(i)^T X(i) + \alpha I \right]^{-1} \tag{39}$$

4 The performance of CDSML on CCLE dataset using different scenarios

Table 1: The performance of CDSML on CCLE dataset using different scenarios including double similarity, single similarity, and no similarity.

	SC	SD	AUC	AUPR	Accuarcy	F1-score	Precision	Recall
No sim	-	-	0.9515	0.977	0.8988	0.9201	0.9462	0.8955
Single similarity	SC_E	-	0.9606	0.9815	0.9064	0.9269	0.9435	0.9113
	SC_M	-	0.9606	0.9815	0.9064	0.9268	0.9441	0.9108
	SC_V	-	0.9606	0.9815	0.9061	0.9265	0.9444	0.9099
	-	SD_S	0.9606	0.9815	0.9061	0.9265	0.9444	0.9099
	-	SD_N	0.9072	0.9354	0.83	0.8637	0.8392	0.8899
	-	SD_P	0.9071	0.9353	0.8292	0.8638	0.8357	0.8943
Double similarity	SC_E	SD_S	0.9514	0.977	0.8989	0.9201	0.9485	0.8934
	SC_E	SD_P	0.9448	0.9718	0.8916	0.9145	0.934	0.8961
	SC_E	SD_N	0.9447	0.9717	0.8911	0.9143	0.9316	0.898
	SC_M	SD_S	0.9514	0.977	0.8988	0.9201	0. 9465	0.8953
	SC_M	SD_P	0.9606	0.9815	0.9063	0.9267	0.944	0.9106
	SC_M	SD_N	0.9605	0.9815	0.9063	0.9267	0.944	0.9106
	SC_V	SD_S	0.9446	0.9716	0.891	0.8714	0.9302	0.8992
	SC_V	SD_N	0.9447	0.9717	0.8913	0.9145	0.9316	0.8983
	SC_V	SD_P	0.9606	0.9815	0.9064	0.9269	0.9437	0.9111

S1 Table represents the complete evaluation of CDSML on CCLE dataset in various double, single, and no similarity scenarios.

5 Comparison of Zhang et al. predictions with imputed labels

A scenario for showing the rationality of imputed labels ($Impute_L^{(Eq.1)}$) is to compare it with Zhang et al. predictions for missing values in binary mode. To this aim, we compared the predictions by Zhang et al.. method with max concentration thresholds and convert them into binary values. The binary predictions y Zhang et al. method is delineated by $Pred_L^{(Zhang)}$. Both $Impute_L^{(Eq.1)}$ and $Pred_L^{(Zhang)}$ are binary vectors. The following metrics compares these two vectors by considering $Pred_L^{(Zhang)}$ as gold standard labels:

- Accuracy: 0.64
- F1-score: 0.77
- Precision: 0.85
- Recall: 0.70
- Jaccard index: 0.6028
- Cosine similarity: 0.77
- Cross entropy: 0.0083

It is not surprising that the computed criteria show high accordance of imputed labels $Impute_L^{(Eq.1)}$ with Zhang et al. predicted labels $Pred_L^{(Zhang)}$ because in section 3.2 of manuscript, we showed that their IC50 values (i.e. $Impute_V^{(Eq.1)}$ and $Pred_V^{(Zhang)}$) are close to each other. The high accordance of imputed labels with predicted labels by Zhang et al. further justifies the rationality of imputed labels.

6 Comparison of Zhang et al. predictions with CDSML predicted labels

In order to compare the Zhang et al. predictions and CDSML predicted labels, we can use the following scenarios:

1. Consider all known values as train and missing values as test data
2. Apply 5 fold cross validation on the known entries and ignore the missing pairs It should be noted that in both scenarios, the predictions of Zahng et al. method were converted to binary labels in order to be comparable with CDSML predicted labels.

6.1 Comparison on missing pairs

We applied CDSML method on GDSC dataset with imputation step and obtained CDSML labels for missing pairs ($Pred_L^{(CDSML)}$). On the other hand, $Pred_L^{(Zhang)}$ labels were obtained according to the explained procedure in previous section. the following metrics compares $Pred_L^{(CDSML)}$ and $Pred_L^{(Zhang)}$, by considering $Pred_L^{(Zhang)}$ as the ground truth labels:

- Accuracy: 0.51
- F1-score: 0.64
- Precision: 0.50
- Recall: 0.88
- Jaccard index: 0.6028
- Cosine similarity: 0.667
- Cross entropy: 0.0348

The evaluated criteria validate that there is a favorable agreement between the predicted labels by CDSML and Zhang et al. method.

6.2 Comparison on known pairs

We evaluated both CDSML and Zhang et al. method using 5-fold cross-validation on known pairs. Then, we convert predicted values by Zhang et al. method into sensitive/resistant labels and compute the classification criteria for them. Table 2 represents the computed criteria for CDSML and Zhang et al. method. It can be seen that CDSML shows better

Table 2: Comparison of CDSML performance with Zhang et al. method performance on known pairs in GDSC. The assessments were done by five-fold cross-validation. The highest value of each criteria is shown in bold.

Method	AUC	AUPR	Accuracy	F1-score	Precision	Recall
CDSML	**0.9157**	**0.9398**	**0.8388**	**0.8715**	**0.8422**	**0.9031**
Zhang et al.	0.4942	0.5516	0.7152	0.7975	**0.9398**	0.6906

performance for predicting classification labels.

7 Supplementary Figure 1

8 Supplementary Figure 2

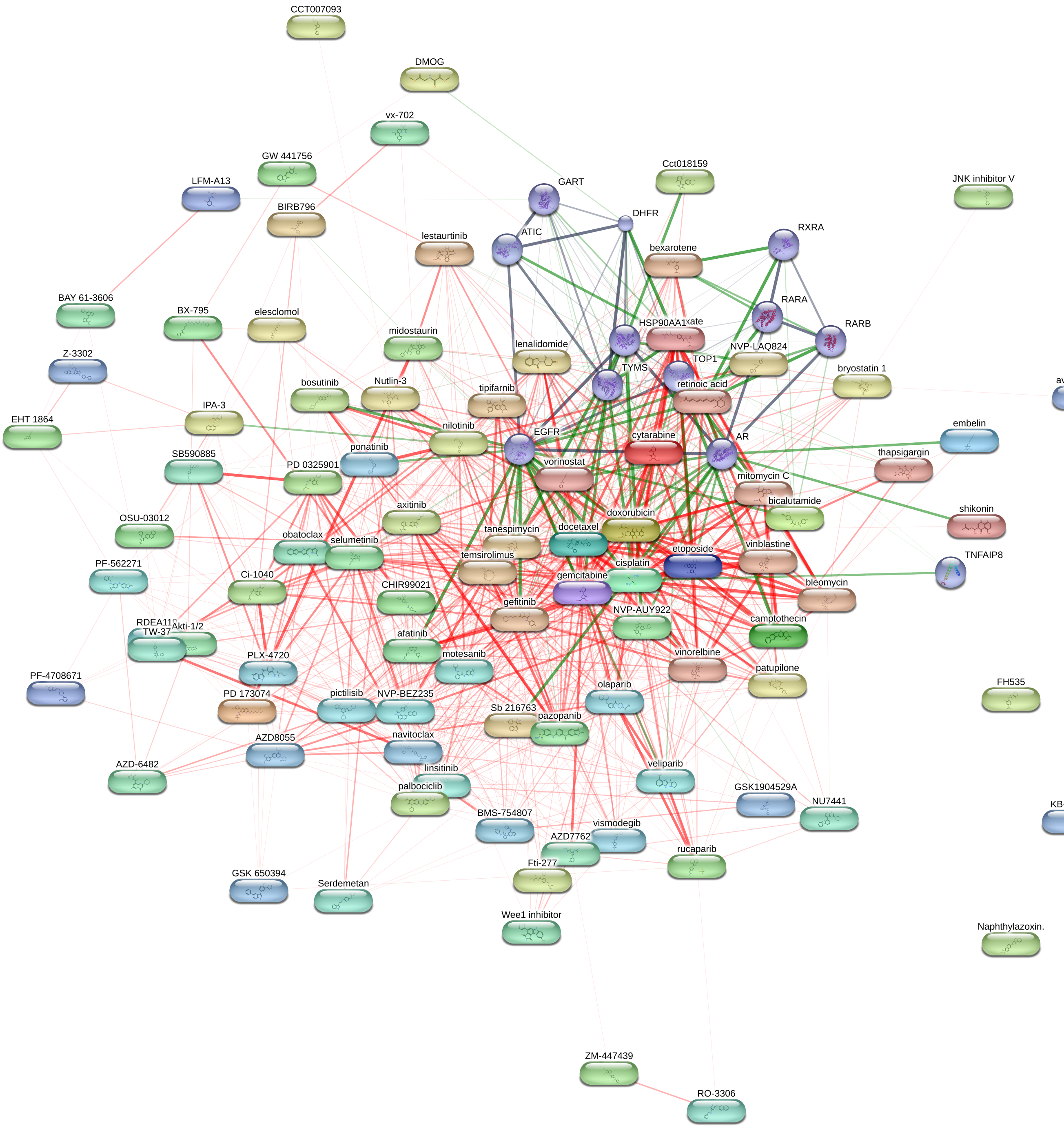

S1 Fig: STiTCH network for drugs in GDSC

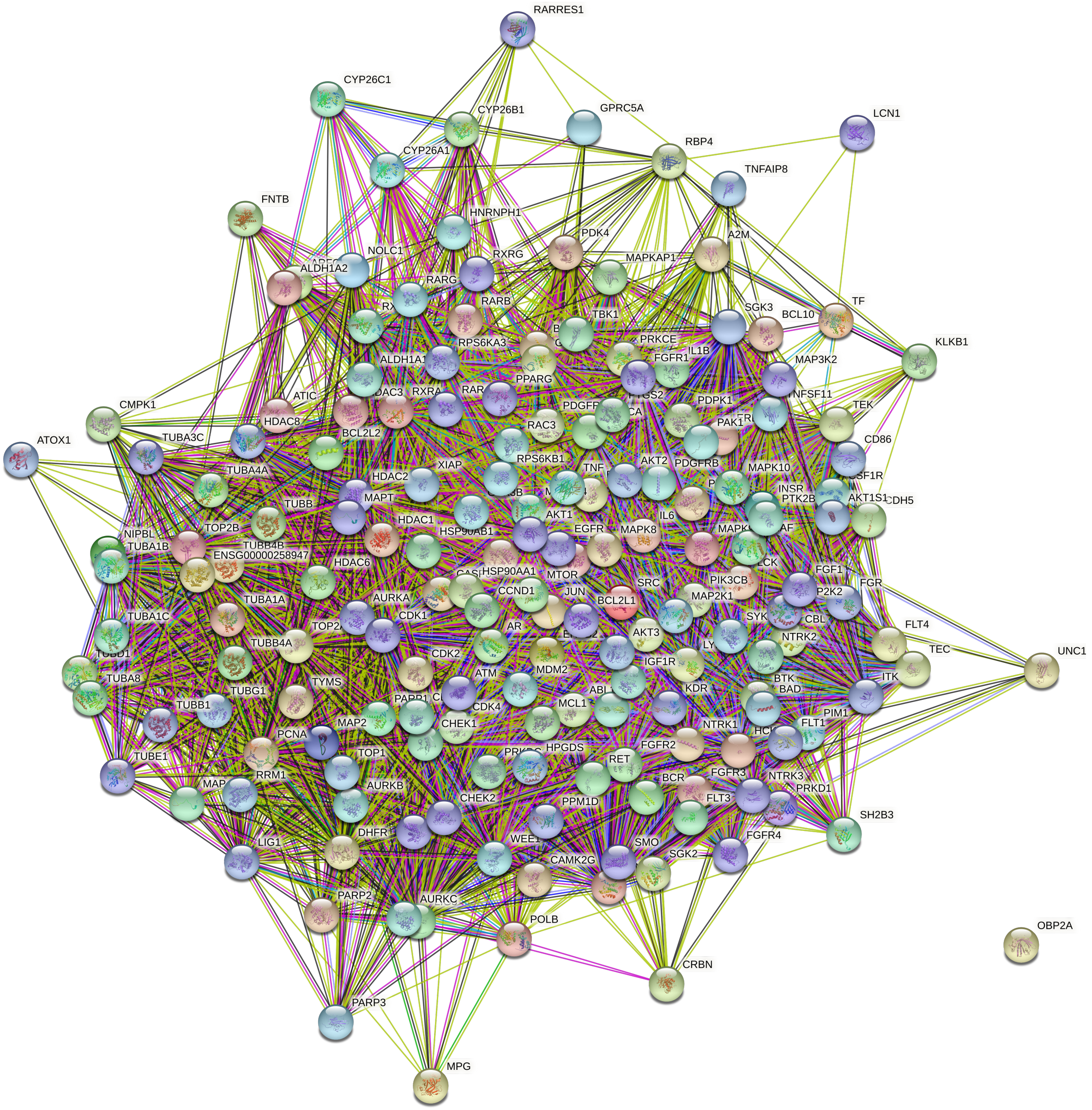

S2 Fig: STRING network for drugs in GDSC
