# Supplementary material for: A computational method for drug sensitivity prediction of cancer cell lines based on various molecular information
Source: PLoS One. 2021 Apr 29;16(4):e0250620. doi: 10.1371/journal.pone.0250620 (PMC8084246; doi:10.1371/journal.pone.0250620)
Supplement: Attachment — Submitted filename: ressponse to reviewers.pdf [file pone.0250620.s003.pdf]

Response to Reviewers

The authors would like to thank the reviewers for their deep and accurate review. Following the reviewers' comments, we have included all the comments point by point, made the required changes in the manuscript, and highlighted them. We hope the revised manuscript will better suit the "PLOS One Journal". The following is our detailed response to the reviewers' remarks.

Reviewer #1:

The authors introduced the CDSML method to predict drug sensitivity of cancer cell lines. They compared it with two types of methods – classification and regression methods on the GDSC dataset. It shows the CDSML performs better than other methods when using the six metrics. This manuscript is well organized and easy to follow. It provides us an effective computational method for drug sensitivity prediction on cancer cell lines. I have the following comments.

We appreciate the reviewer for the positive impression on our manuscript. We respond to the comments point by point in the following.

***Major**:*

1. In the objective function, it seems that you could solve this problem without dealing with missing values. Because in the first term in Eq.(8), one could ignore the drug-cell line pair for which B(i,j) value is missing.

We thank your helpful suggestion that helps to extend the application of our method. We altered the loss function as the following:

$$Loss = \frac{1}{2}\sum_{i,j \notin Missing} (B(i,j) - X(i)Y(j)^T)^2 + \frac{\alpha}{2}\left(\sum_i ||X(i)||^2 + \sum_j ||Y(j)||^2\right) + \frac{\beta}{2}\left(\sum_{i,j} ||X(i) - X(j)||^2\ SC(i,j) + \sum_{i,j} ||Y(i) - Y(j)||^2 SD(i,j)\right)$$

Consequently, according to Eq. 13 and 14 (Newton method), we altered $\nabla_{X(i)^{(k)}}\text{Loss}$ and $\nabla^2_{X(i)^{(k)}}\text{Loss}$ for updating latent vectors according to the following formula.

$$\nabla_{X(i)^{(k)}} Loss = \sum_{i,j \notin Missing} (X(i)Y(j)^T - B(i,j))Y(j) + \alpha X(i) + \beta \sum_{j} (X(i) - X(j))SC(i,j) - \beta \sum_{j} (X(j) - X(i))SC(j,i)$$

$$\nabla^2_{X(i)^{(k)}} Loss = \sum_{i,j \notin Missing} Y(j)^T Y(j) + \alpha I + \beta \sum_{j} (SC(i,j) + SC(j,i))I$$

Therefore, the latent vector $X(i)^{(k)}$ is updated according to the following formula:

$$X(i)^{(k+1)} = \left[\sum_{i,j \notin Missing} B(i,j)Y(j) + \beta \sum_{j} \big(SC(i,j) + SC(j,i)\big)X(i)^{(k)} \right] \left[\sum_{ii,j \notin Missing} Y(j)^T Y(j) + \alpha I + \beta \sum_{j} \big(SC(i,j) + SC(j,i)\big)I \right]^{\{-1\}}$$

Similarly, the latent vector $Y(j)^{(k)}$ is updated according to the following formula:

$$Y(j)^{(k+1)} = \left[\sum_{i,j \notin Missing} B(i,j)X(j) + \beta \sum_{i} \big(SD(i,j) + SD(j,i)\big)Y(j)^{(k)} \right] \left[\sum_{ii,j \notin Missing} X(i)^T X(i) + \alpha I + \beta \sum_{i} \big(SD(i,j) + SD(j,i)\big)I \right]^{\{-1\}}$$

We amended the implementation such that the new code is capable of handling the response matrix with or without missing values. The missing values must be indicated by the null sign "NA". It should be noted that using these formulas also works for the response matrix without missing. For example, when the missing values are imputed, the set of $Missing$ is empty; therefore, in that case the loss terms are calculated for all pairs.

c.f. (reference in the revised materials)

– GitHub repository: https://github.com/ fahmadimoughari/CDSML

– Revised manuscript: Section 2.5.1 on pages 10, 11

–Supplementary Materials: Section 2 on pages 4, 5

2. Following the above question, could the author compare the predicted values for missing pairs and the imputing values by Eq.(1) to show the reasonability of the imputing method in the paper.

We think this is an excellent suggestion. We executed CDSML on the binary response matrix with missing values (not applying imputation procedure), while considering all known pairs as training samples and all missing pairs as test samples. The estimated labels (1:sensitive, 0:resistant) of CDSML for these pairs are considered as $Pred_L^{(CDSML)}$ vector (the index "L" shows that the vector consists of binary labels).

On the other hand, we computed the imputed values by Eq. 1 in the paper and then converted these values to the labels by comparing imputed IC50 values with max concentration thresholds of drugs. The imputed values less than the threshold were considered as 1 and the pairs with imputed values greater than threshold were considered as 0. We denote these imputed labels by $Impute_L^{(Eq.1)}$ vector.

There are 1479 cell line-drug pairs in GDSC where their response values are missed. Therefore, the length of $Pred_L^{(CDSML)}$ and $Impute_L^{(Eq.1)}$ vectors are 1479. It is noteworthy that $Pred_L^{(CDSML)}$ contains 726 sensitive labels, while $Impute_L^{(Eq.1)}$ vector contains 1041 sensitive labels. Several metrics were computed to compare $Pred_L^{(CDSML)}$ and $Impute_L^{(Eq.1)}$ vectors.

Since both vectors are binary, computing Accuracy, F1-score, Precision, and Recall in addition to the Jaccard index, cosine similarity, and binary cross-entropy are meaningful and can give us an extensive comparison of these two vectors.

Note that we need to compute true positive, true negative, false positive, and false negative samples to compute the Precision and Recall. Therefore, we must consider one of $Pred_L^{(CDSML)}$ or $Impute_L^{(Eq.1)}$ as the ground truth labels. The following criteria are calculated based on considering $Pred_L^{(CDSML)}$ vector as the ground truth.

- Accuracy: 0.718
- F1-score: 0.764
- Precision: 0.9297

- Recall: 0.6484
- Jaccard index: 0.6181
- Cosine similarity: 0.7764
- Cross-entropy: 0.0065

It should be mentioned that the Precision calculated by considering $Pred_L^{(CDSML)}$ as the ground truth is equal to the Recall value when we consider $Impute_L^{(Eq.1)}$ as the ground truth. Moreover, the Recall calculated by considering $Pred_L^{(CDSML)}$ as the ground truth is equal to the Precision value when we consider $Impute_L^{(Eq.1)}$ as the ground truth. However, Accuracy and F1-score do not change when we consider each of $Pred_L^{(CDSML)}$ or $Impute_L^{(Eq.1)}$ as the ground truth.

Both the computed criteria using each of $Pred_L^{(CDSML)}$ and $Impute_L^{(Eq.1)}$ as the ground truth are highly favorable. Too little value of cross-entropy as well as the high values of other metrics verifies the high accordance of imputed and predicted labels and justifies the reasonability of imputed values.

c.f. (reference in the revised materials)

– Revised manuscript: Section 2.5.1 on pages 10, 11 and Section 3.2 on pages 13, 14.

3. The idea of the method in this paper "Predicting Anticancer Drug Responses Using a Dual-Layer Integrated Cell Line-Drug Network Model" is very similar to the imputing way (Eq.(1)) for missing values, could you compare CDSML with it?

We thank the reviewer for providing this valuable reference. The idea in the provided reference (Zhang et al.) for predicting IC50 values is similar to Eq. 1 in our paper. However, there are some differences between these two strategies. To name one difference, we considered only k-nearest neighbor samples for predicting imputed values in our paper, while Zhang et al. considered the whole known samples for predicting IC50. The overall attitude is very similar. We compared CDSML with Zhang et al. method in five scenarios.

1. First scenario

We first compared the imputed IC50 values computed by Eq. 1 ($Impute_V^{(Eq.1)}$) with the predicted IC50 values by Zhang et al. method ($Pred_C^{(Zhang)}$) on the missing pairs (the index "C" shows that the vectors consist of continuous values).

Since both $Impute_C^{(Eq.1)}$ and $Pred_C^{(Zhang)}$ vectors are continuous, we computed continuous metrics such as root-mean-square error (RMSE), normalized root-mean-square error (NRMSE), and mean absolute error (MAE) to compare the vectors. The computed criteria are as follows:

- RMSE : 0.04369
- NRMSE_(Impute): 0.0065
- NRMSE_(Zhang): 0.0081
- MAE: 1.45

Where NRMSE_(Impute) and NRMSE_(Zhang) are computed by considering $Impute_C^{(Eq.1)}$ and $Pred_C^{(Zhang)}$ as true values, respectively. Based on low values of RMSE, NRMSE, and MAE, it is validated that the predicted values by Zhang et al. are so close to the imputed values by Eq. 1.

2. Second scenario

Moreover, we converted the imputed values by Eq. 1 and Zhang et al. predicted values to sensitive/resistant labels by comparing to max concentration thresholds. Then, we compared the obtained binary vectors of imputed labels ($Impute_L^{(Eq.1)}$) and Zhang et al. predicted labels ($Pred_L^{(Zhang)}$) using binary metrics reported as follows:

- Accuracy: 0.64
- F1-score: 0.77
- Precision: 0.85
- Recall: 0.70
- Jaccard index: 0.6028
- Cosine similarity: 0.77
- Cross entropy: 0.0083

It is not surprising that the computed criteria show high accordance of imputed labels by Eq.1 ($Impute_L^{(Eq.1)}$) with Zhang et al. predicted labels ($Pred_L^{(Zhang)}$) because in the previous scenario, we showed that their IC50 values (i.e. $Impute_C^{(Eq.1)}$ and $Pred_C^{(Zhang)}$) are close to each other.

3. Third scenario

The third scenario is to compare the predicted labels by CDSML ($Pred_L^{(CDSML)}$) and predicted labels by Zhang et al. method ($Pred_L^{(Zhang)}$) for missing values. To this aim, we applied CDSML on a non-imputed response matrix and predicted sensitive/resistant labels for missing pairs. Computed metrics are as follows:

- Accuracy: 0.51
- F1-score: 0.64
- Precision: 0.50
- Recall: 0.88
- Jaccard index: 0.6028
- Cosine similarity: 0.667
- Cross entropy: 0.0348

The evaluated criteria validate that there is a favorable agreement between the predicted labels by CDSML and Zhang et al. method.

4. Forth scenario

Another way to compare CDSML results with Zhang et al. predictions is to ignore the missing values and to assess the methods using 5-fold cross-validation on known pairs. The following tables represent the computed criteria for CDSML and Zhang et al. method in classification mode.

Method	*Dataset*	*AUC*	*AUPR*	*Accuracy*	*F1-score*	*Precision*	*Recall*
CDSML	**GDSC**	**0.9157**	**0.9398**	**0.8388**	**0.8715**	0.8422	**0.9031**
Zhang et al.	GDSC	0.4942	0.5516	0.7152	0.7975	**0.9398**	0.6906

It can be seen that CDSML shows better performance for predicting classification labels.

5. Fifth scenario

As the last validation, we used the Zhang et al. predicted IC50 values ($Pred_C^{(Zhang)}$) instead of using Eq. 1 in order to fill in the missing values of the response matrix and then apply the CDSML manifold learning on the obtained matrix. Let us call this version as CDSML-Zhang. We compared CDSML-Zhang with CDSML using a stratified 5-fold cross-validation. The evaluation metrics are as follows:

Method	*AUC*	*AUPR*	*Accuracy*	*F1-score*	*Precision*	*Recall*
CDSML	**0.917**	**0.94**	**0.839**	**0.871**	**0.843**	**0.903**
CDSML-Zhang	0.912	0.937	0.836	0.870	0.84	0.896

The results are very similar; however, CDSML using Eq. 1 for imputing missing values leads to better results.

To sum up, all validation scenarios confirm that the imputed values using Eq. 1 are reasonable and lead to the improvement in results.

c.f. (reference in the revised materials)

– Revised manuscript: Section 3.3 on pages 14, 15.

-Supplementary Materials: Sections 5 and 6 on pages 9, 10.

4. Regarding to the objective function, how to ensure the entries in the estimated matrix B range from 0 to 1? There is no constraint about the scale and sign for values of X(i) and Y(j).

Thanks for your consideration. There is no constraint to guarantee that the predicted scores by CDSML lie within the range of [0, 1]. We checked all the predicted scores and observed that all predicted scores ranged from 0 to 1, while there is no specific constraint for it. This is due to the fact that the estimated scores should have minor differences from real binary labels according to the first term in the loss function. Therefore, there is a constraint to compel the predicted scores to be very close to 0 and 1.

Moreover, it should be noted that there is no necessity or requirement to estimate the values in the range of [0, 1] because the estimated scores are eventually converted to binary labels using a threshold. This threshold is determined using Precision-Recall Curve and it does not require probabilistic values. Furthermore, the AUC and AUPR calculations do not require probabilistic estimations. In sum, if the estimated scores do not lie in the range of [0, 1], nothing goes wrong.

We thank the reviewer to note that the estimated scores may not be probabilistic. Accordingly, we changed some terms used in the paper. For example, we changed "sensitivity and resistance

probability" to "sensitivity and resistance scores", respectively and changed the "estimated probability" to "estimated score".

5. In Table 2, CDSML performs similarly when using gene expression, mutation and CNV to calculate cell line similarity. How about estimating B(i,j) without the matrix SC in the objective function? It will test the power of cell line similarity matrix SC and drug similarity matrix SD when prediction.

We used the reviewer's suggestion and extended the application of implemented code based on this idea such that it can ignore cell line and/or drug similarities. The final implementation is able to handle missing values in response matrix and has optional settings for ignoring any similarities. We altered the loss function as the following.

If only SC is ignored, the loss function will be changed to the following:

$$Loss^{(SD)} = \frac{1}{2} \sum_{i,j \notin Missing} (B(i,j) - X(i)Y(j)^T)^2 + \frac{\alpha}{2}\left(\sum_i ||X(i)||^2 + \sum_j ||Y(j)||^2 \right) + \frac{\beta}{2}\left(\sum_{i,j} ||Y(i) - Y(j)||^2 SD(i,j) \right)$$

If only SD is ignored, the loss function will be changed as the following:

$$Loss^{(SC)} = \frac{1}{2} \sum_{i,j \notin Missing} (B(i,j) - X(i)Y(j)^T)^2 + \frac{\alpha}{2}\left(\sum_i ||X(i)||^2 + \sum_j ||Y(j)||^2 \right) + \frac{\beta}{2}\left(\sum_{i,j} ||X(i) - X(j)||^2 \; SC(i,j) \right)$$

And if both SC and SD are ignored, the loss function will be changed to the following form:

$$Loss^{(No\ sim)} = \frac{1}{2} \sum_{i,j \notin Missing} (B(i,j) - X(i)Y(j)^T)^2 + \frac{\alpha}{2}\left(\sum_i ||X(i)||^2 + \sum_j ||Y(j)||^2 \right)$$

It is notable that, in each scenario, the formula for updating latent matrices will be adjusted based on the related loss functions.

We evaluated CDSML in three scenarios:

1. *Double similarity*: Using both SC and SD
2. *Single similarity*: Using only one of SC or SD
3. *No similarity*: Using no similarities

We used stratified 5-fold cross-validation to compare all scenarios. The evaluation criteria are shown in the following table. It should be noted that according to another reviewer's comments, we computed two other types of SD in the revised version. It should be mentioned that we applied various similarity fusion or integration techniques, including linear, network-based, covariance-based, and propagation methods, to integrate the similarity matrices; however, the improvement of the results was not significant enough to add such complexity to the model.

SCENARIO	SC	SD	AUC	AUPR	ACCURACY	F1-SCORE	PRECISION	RECALL
NO SIM	**Not used**	Not used	0.8542	0.8846	0.7812	0.8291	0.7706	0.8974
SINGLE SIM	EXP	Not used	0.9147	0.9372	0.8359	0.8653	0.841	0.891
	MUT	Not used	0.9148	0.9373	0.8356	0.8652	0.8401	0.892
	CNV	Not used	0.9071	0.9353	0.8288	0.8636	0.8346	0.8948
	**Not used**	CHEM	0.9071	0.9353	0.8288	0.8636	0.8346	0.8951
	**Not used**	NET	0.9072	0.9354	0.83	0. 8637	0. 8392	0. 8899
	**Not used**	PPI	0.9071	0.9353	0.8292	0.8638	0.8357	0.8943
DOUBLE SIM	EXP	CHEM	**0. 9158**	0. 9373	**0. 8388**	0. 8714	**0. 8426**	0. 9026
	EXP	PPI	0. 9157	0. 9373	0. 8354	0. 8714	0. 8423	0. 903
	EXP	NET	0. 9157	**0. 9398**	**0. 8388**	**0. 8715**	0. 8422	**0. 9031**
	MUT	CHEM	0. 9148	0. 9373	0. 8357	0. 8652	0. 8402	0. 8918
	MUT	PPI	0. 9147	0. 9373	0. 8354	0. 8652	0. 8392	0. 8929
	MUT	NET	0. 9147	0. 9373	0. 8350	0. 8652	0. 8388	0. 8949
	CNV	CHEM	0. 9157	0. 9397	**0. 8388**	0. 8714	**0. 8426**	0. 9026
	CNV	NET	0. 9157	**0. 9398**	0. 8387	0. 8714	0. 8424	0. 9028
	CNV	PPI	0. 9147	0. 9372	0. 8355	0. 8653	0. 8390	0. 8934

We discuss each of three scenarios in the following.

- **Discussing "No Similarity" scenario**:

The results in the first row of the above table indicate that CDSML gained high performance even using no similarities. CDSML performance using no similarity confirmed that the matrix factorization used in CDSML is efficient itself without using other extra information. The CDSML results using no similarity outperform most of the state-of-the-art methods mentioned in the paper.

- **Discussing "Single Similarity" scenario**:

Based on this table, one can conclude that using a single similarity matrix makes about 6% improvements of all criteria compared to the case of no similarities. Moreover, the performance of CDSML on various types of SC or various types of SD leads to almost equal performance, i.e. the calculated criteria for all executions on a single similarity matrix are the same. For example, using only the copy number variation similarity of cell lines leads to AUC of 0.9071 and AUPR of 0.9353, while using solely the PPI similarity of drugs also leads to the same values of AUC and AUPR. Therefore, the impact of all similarity matrices on the CDSML performance are almost equal.

- **Discussing "Double Similarity" scenario**:

Moreover, by comparing the results in single similarity and double similarity scenarios, it can be inferred that the performance of CDSML using both SC and SD makes subtle improvement in comparison using single similarity.

c.f. (reference in the revised materials)

– GitHub repository: https://github.com/ fahmadimoughari/CDSML

– Revised manuscript: Section 2.5.2 on pages 11, 12 and Section 3.2 on pages 12, 13

-Supplementary Materials: Sections 3 and 4 on pages 5 to 8.

6. When performing the 5-fold CV procedure, did you make sure that the percentage of positive and negative cases for training dataset is the same with the testing dataset? I think this is important for classification problem. Under your threshold C_{max}, please provide the percentage of sensitive drug-cell line pairs.

We admit that using stratified cross-validation can evaluate the methods in a way that is more reliable. We modified the implementation of CDSML and evaluated using a stratified 5-fold cross-validation in order to ensure that the fraction of positive samples over negative samples is almost equal in train and test data. Accordingly, we evaluated all other methods using the same strategy of stratified 5-fold cross-validation in order to conduct fair comparisons. We updated all the evaluated criteria in all tables of the paper based on the modified evaluations.

To answer the second part of this comment, we should mention that when max concentration is used to label IC50 values, the number of sensitive values in GDSC and CCLE are as follows:

- Among 54,390 pairs in GDSC, about %59 of pairs, which is 32,164 pairs were labeled as sensitive
- %65 of pairs in CCLE are labelled as sensitive which is 4,254 out of 6534 pairs.

c.f. (reference in the revised materials)

– GitHub repository: https://github.com/ fahmadimoughari/CDSML

– Revised manuscript: The first paragraph in Section 2.4 on page 10, the last paragraph in Section 2.2.2 on page 5, and all values reported in Tables 2, 6, 8 and 9.

7. CCLE is another commonly used dataset for drug sensitivity prediction study. It is better to apply the methods on this dataset to show its efficiency.

As suggested by the reviewer, we computed various types of similarities for cell lines and drugs in CCLE. Moreover, we restricted CCLE dataset to the drugs that their max concentration values are available. This restriction is similar to what is done in the previous studies (such as RefDNN). Among 24 drugs in CCLE, 18 drugs were in common with GDSC, and so their max concentration values are available. We evaluated all methods on CCLE dataset using stratified 5-fold cross-validation. The results on CCLE are presented in the following table.

The results on CCLE also show that CDSML outperforms other methods and further confirm the efficiency of the proposed method.

c.f. (reference in the revised materials)

– GitHub repository: https://github.com/ fahmadimoughari/CDSML

– Revised manuscript: Section 2.1 on page 3 and Tables 6, 8 and 9.

–Supplementary Materials: Sections 4 on pages 8.

	**METHOD**	**AUC**	**AUPR**	**ACCURACY**	**F1-SCORE**	**PRECISION**	**RECALL**
**CLASSIFICATION METHODS**	CDSML	**0.9514**	**0.977**	**0.8989**	**0.9201**	0.9485	0.8934
	RefDNN	0.6951	0.8200	0.6796	0.7825	0.7109	0.8713
	DSPLMF	0.8148	0.6944	0.7405	0.6667	0.5906	0.7668
	HNMPRD	0.5817	0.7228	0.6527	0.7889	0.6528	**0.9967**
**REGRESSION METHODS**	SRMF	0.4539	0.6266	0.6966	0.8005	0.6998	0.9351
	CaDRReS	0.4389	0.6732	0.7133	0.8138	0.7048	0.9625
	CDCN	0.4262	0.6204	0.8608	0.8824	**0.9796**	0.8029
	ADRML	0.4257	0.6229	0.5473	0.6703	0.6373	0.7071
**MACHINE LEARNING MODELS**	GNB	0.9111	0.9523	0.842	0.8779	0.8914	0.8675
	LR	0.9435	0.9651	0.8811	0.9189	0.948	0.8916
	RF	0.9494	0.9632	0.8884	0.9193	0.9463	0.8939
	SVM	0.9444	0.9694	0.8867	0.9192	0.9478	0.8924
	MLP	0.9376	0.9671	0.8744	0.9146	0.9624	0.872
	Ada	0.9475	0.9609	0.8878	0.9118	0.9332	0.8924
	KNN	0.936	0.9653	0.8788	0.9116	0.907	0.8843

Minor:

1. In the Abstract, "achieving AUC of 0.92%, xxxxx 0.94%, xxxx". The percent sign % should be removed.

Thanks for your consideration. We edited the abstract and removed the percent signs.

c.f. (reference in the revised materials)

– Revised manuscript: Abstract on page 1

2. Please check the compared method names, and make sure they are the same in the manuscript, for example, SRMF and SRMf.

Thanks for pointing this out. We went through the manuscript and unified the method names across the manuscript.

3. Could you provide the values for the parameters \alpha, \beta and the latent number k in Eq.(8) in the Table 4? And in Table 4, which metric do you use to select the best hyper-parameters for these methods since there are 6 metrics in Table 5?

Thanks for mentioning this issue. We added the values for CDSML hyper-parameters to table 5 (the numbers of tables are changed in the revised manuscript, so the current table 5 is the previous table 4). Moreover, the AUC criterion is used to determine the best values of hyper-parameters because AUC does not depend on the threshold value and evaluates the models more comprehensively.

c.f. (reference in the revised materials)

– Revised manuscript: Section 3.1 on page 12, Lines 503, 504 and the last row in Table 7.

4. The figures in this paper have very low resolution, please improve that.

We thank the reviewer for this comment. We enhance the quality of figures. However, there is an issue with the Journal pdf maker. I uploaded all the high resolution files for the figures separately. But, in the pdf made by journal, the quality of figures are lowered. By the way, I think you can download the high-resolution figures separately.

5. The section number is strange, 0.1, 0.2 ….

We edited the section numbers in the revised manuscript

c.f. (reference in the revised materials)

– Revised manuscript: All section numbers

6. I downloaded the data from the github website, all the entries in the table "SC_CopyNumberVariation.csv" are zero. Did you upload a wrong table?

We appreciate the reviewer for notifying this important issue. We have uploaded the similarity matrices after applying symmetric Laplacian normalization. We noticed that SC based on copy number variation is a meaningful matrix but applying normalization on it transforms it to a completely zero matrix. This is due to the fact that SC based on copy number variation contains negative entries, which lead to null values in the computation of $D^{-\frac{1}{2}}$ matrix and the normalization procedure eventually leads to a completely zero matrix. Since the similarity matrices were computed based on the Pearson correlation coefficient or the Jaccard index, their values lies in the range of [-1,1]. Therefore, we applied a standardization technique in order to ensure that all similarity values lie in [0, 1] range. Assume $S(i,j)$ be an entry in a similarity matrix. The standardized value $\hat{S}(i,j)$ is computed as the following:

$$\hat{S}(i,j) = \frac{S(i,j) + 1}{2}$$

Since this standardization is a linear transform, it does not alter the sorting of the distance between samples.

Then, we applied the symmetric Laplacian normalization on the standardized that similarity matrices. It should be noted that the normalized matrices are meaningful and are not completely zero using this version. Moreover, we uploaded the modified versions of similarity matrices after applying the standardization and normalization steps on Github repository.

c.f. (reference in the revised materials)

– GitHub repository: https://github.com/ fahmadimoughari/CDSML

– Revised manuscript: Section 2.2.4 on page 7

Reviewer #2:

1. Avoid using abbreviation in abstract

Thank you for pointing this out. We edited the abstract and removed all the abbreviations.

c.f. (reference in the revised materials)

– Revised manuscript: Abstract on page 1

2. Performance gain in the new method (CDSML) compared to the old one (ADRML) should be made clear

We appreciate the reviewer's comments, which leads to considering ADRML results in this paper. It should be noted that CDSML uses sensitive/resistant labels of the training pairs as the input. CDSML applies manifold learning on the binary response matrix and estimates the sensitivity scores as the result. To convert the sensitivity scores obtained by CDSML into sensitive/resistant labels, we applied the best threshold based on the elbow-point of the Precision-Recall curve.

On the other hand, ADRML is a regression method, which receives the Ic50 values of training pairs as the input. ADRML applies manifold learning on the real-valued (IC50) response matrix and estimates the IC50 values as the result. Eventually, to compare the ADRML performance with classification methods, we applied the max concentration threshold on the estimated values to convert them into estimated sensitive/resistant labels.

Therefore, the difference between the CDSML and ADRML methods are their input response matrix and the final thresholds on the model predictions.

According to the procedure that we applied to the regression methods for comparing them with classification methods, we performed ADRML on CCLE and GDSC datasets in regression mode using IC50 values. Then, we converted the predicted IC50 values into sensitive/resistant labels using max concentration thresholds. The evaluation metrics using stratified 5-fold cross-validation of ADRML and CDSML using gene expression cell line similarity and chemical substructure similarity of drugs are provided in the following table.

Method	*Dataset*	*AUC*	*AUPR*	*Accuracy*	*F1-score*	*Precision*	*Recall*
CDSML	**GDSC**	**0.917**	**0.94**	**0.839**	**0.871**	**0.843**	0.903
ADRML	GDSC	0.4077	0.5096	0.7501	0.8177	0.7189	**0.9481**
CDSML	CCLE	**0.9514**	**0.977**	**0.8989**	**0.9201**	**0.9485**	**0.8934**
ADRML	CCLE	0.4257	0.6229	0.5473	0.6703	0.6373	0.7071

One can conclude that CDSML significantly outperforms ADRML. Since CDSML uses a binary response matrix, it can be inferred that using a binary response matrix as the initial input of manifold learning leads to a more reliable classification of cell line-drug pairs into sensitive/resistant categories.

c.f. (reference in the revised materials)

– Revised manuscript: Section 3.5 on page 17 and 18.

3. Author should clarify what is the novelty in this work compared to the previous approach (i.e. ADRML).

We acknowledge the reviewer's comments. We should have mentioned the novelty of this paper more clearly. The contributions of this paper are as follows:

- The previous method (ADRML) is a regression method and uses IC50 values for training the model. We changed this method in this paper in such a way that can be used in the classification form with high efficiency. The results provided in the table 9 evidently show that performing ADRML and just applying a threshold on the predicted IC50 values does not lead to satisfactory performance in the classification area. Since suggesting efficient drugs for patients in precision medicine uses the sensitivity of patients to the anti-cancer drugs, it is more essential to predict the sensitivity or resistance label instead of the response values. Therefore, the classification problem has a higher importance than the regression problem in this area. In this paper, we have shown that applying max concentration threshold on the inputs and predicting sensitive/resistant labels using manifold learning leads to more reliable sensitivity prediction.
- Moreover, it is helpful to figure out the importance of each cell line or drug similarities on the performance of the classification model. In this paper, we fully investigate the effect of using no similarity, using only the cell line similarity, using only the drug similarity, and using both the drug and cell line similarities on the prediction performance.
- Numerous previous studies have proposed efficient methods for predicting drug responses using regression or classification models. It is highly efficient to set up a framework for comparing all regression and classification methods in a common setting and using fair comparison. We provide a framework to convert the regression models into classification models and compare all methods in classification mode. To this aim, we applied the max concentration threshold on the predicted IC50 values in order to convert the predicted values into predicted sensitivity/resistance labels.
- The proposed method in this paper has the capability of handling missing values with or without imputation strategy. Moreover, the implemented code is able to perform the proposed method using no similarity, one type of similarity, or both cell line and drug similarities. The implemented code has the capability to choose the suitable loss function and

optimization procedure based on the options that the user determined for the usage of similarity information.

- Several previous papers have used an imputation approach based on nearest neighbors to impute the missing values in the response matrix or the feature matrices. The reasonability of using this procedure for imputing missing values was not fully validated in the previous studies. Here, we confirmed the reliability of using this procedure for imputing missing values using four different scenarios.
- Since the algebraic and spectral characteristics of matrices used in manifold learning influence the convergence of the model, we proposed to use the combination of standardization and normalization in this paper in order to handle the negative similarity values and transforming the similarities to more informative matrices with desirable characteristics. Using the combination of standardization and symmetric Laplacian normalization is novel.
- Furthermore, we computed two novel similarities for drugs based on the maximum matching in target PPI network obtained from STRING database and the computed Jaccard index in STiTCH network. These two types of similarities for drugs are fully described in the response to the subsequent comments and in the revised manuscript. It should be noted that the proposed idea for computing these drug similarities leads to the calculation of highly informative and comprehensive similarities for drugs using the combination of various types of information. Since several drugs in GDSC are not FDA-approved, the information about these drugs is not rich. Therefore, introducing new similarities for these anti-cancer drugs is productive. The newly introduced drug similarities may help future studies in this field and other fields related to drug discovery.

c.f. (reference in the revised materials)

– Revised manuscript: Section 1 on pages 2 and 3, and Section 4 on pages 20 to 22.

4. Writing should be improved in several places, e.g. several short sentences should be paraphrased in a better way.

We are grateful for your consideration. We went through the paper, revised it carefully, and enhanced its writing style as far as we can. Moreover, we used Grammarly website, Quil bot, and specialists' comments to improve the English writing of the paper.

5. Data preprocessing steps should be clearly mentioned in the method section.

The pre-processing steps include four steps:

1. Imputing missing values
2. Converting IC50 values into binary categories
3. Similarity calculation
4. Standardization and normalization

We organized the pre-processing steps in the revised manuscript more clearly and added further explanation and discussion about the assumptions and methodologies to clarify the steps better.

c.f. (reference in the revised materials)

– Revised manuscript: Pages 3-8

6. Including two types of cell-line similarity profiles, authors have used only one type of drug similarity profiles based on chemical substructure. I would suggest to include other types of multi-modal drug similarities, e.g. target protein sequence similarity, target pathway similarity and functional similarity would affect the overall perfomance. See Azad et. al, 2020 [Brief. in Bioinf.] for reference.

We highly appreciate your constructive comment for considering various types of drug similarities. We searched a lot to find various types of features for drugs in GDSC; however, we failed to find adequate information for drugs in GDSC from available databases such as DrugSimDB, Chemical Checker, MeSHDD, and DrugSpaceX. Some of the drugs in GDSC are not FDA approved, and their information is not available in the mentioned databases. Moreover, the available information in these databases has numerous missing values; therefore, there acquired features from these resources were not adequate to train the model.

Fortunately, STRING and a STiTCH databases provides comprehensive information about GDSC drugs. We used these databases to introduce two new drug similarities:

1. **STiTCH network similarity**

First, we obtained STiTCH network for drugs in CCLE and GDSC. The STiTCH network for CCLE is shown in the following figure. The oval nodes in this figure represent the drugs in CCLE and other adjacent drugs, while the circle nodes indicate the neighbor proteins. We computed drug similarities based on STiTCH network SD_N according to the following Equation:

$$SD_N(d_i, d_j) = \frac{|N_i \cap N_j|}{|N_i \cup N_j|}$$

where N_i represents the immediate neighbors (both protein neighbors and chemical neighbors) of drug d_i. Therefore, the jaccard index of the neighbor nodes in STiTCH network is defined as SD_N similarity of drugs.

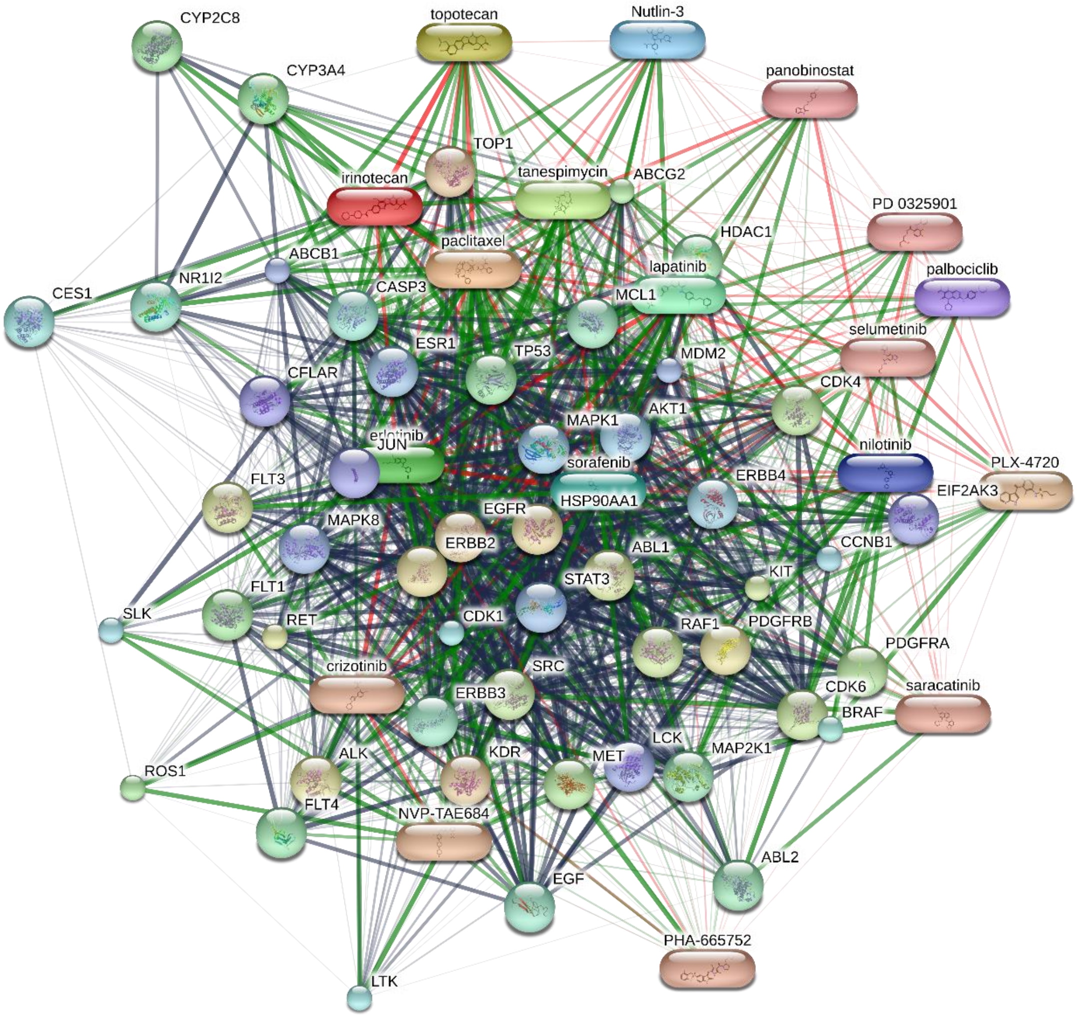

2. Target PPI similarity

In addition to the above drug similarity, we defined another similarity based on protein-protein interaction (PPI) network of target proteins. To this aim, we obtained drug targets and considered the target proteins for all drugs in the dataset as the *target protein set*. Afterward, we acquired PPI network for the *target protein sets* from STRING database. The following figure represents the

STRING PPI network for *target protein set* of CCLE drugs. It is noteworthy that STRING database uses various types of data such as Gene ontology terms, pathways experimental evidence, and text mining features to compute the interactions between proteins. Hence, the weights of edges in STRING PPI network is the combination of various evidence.

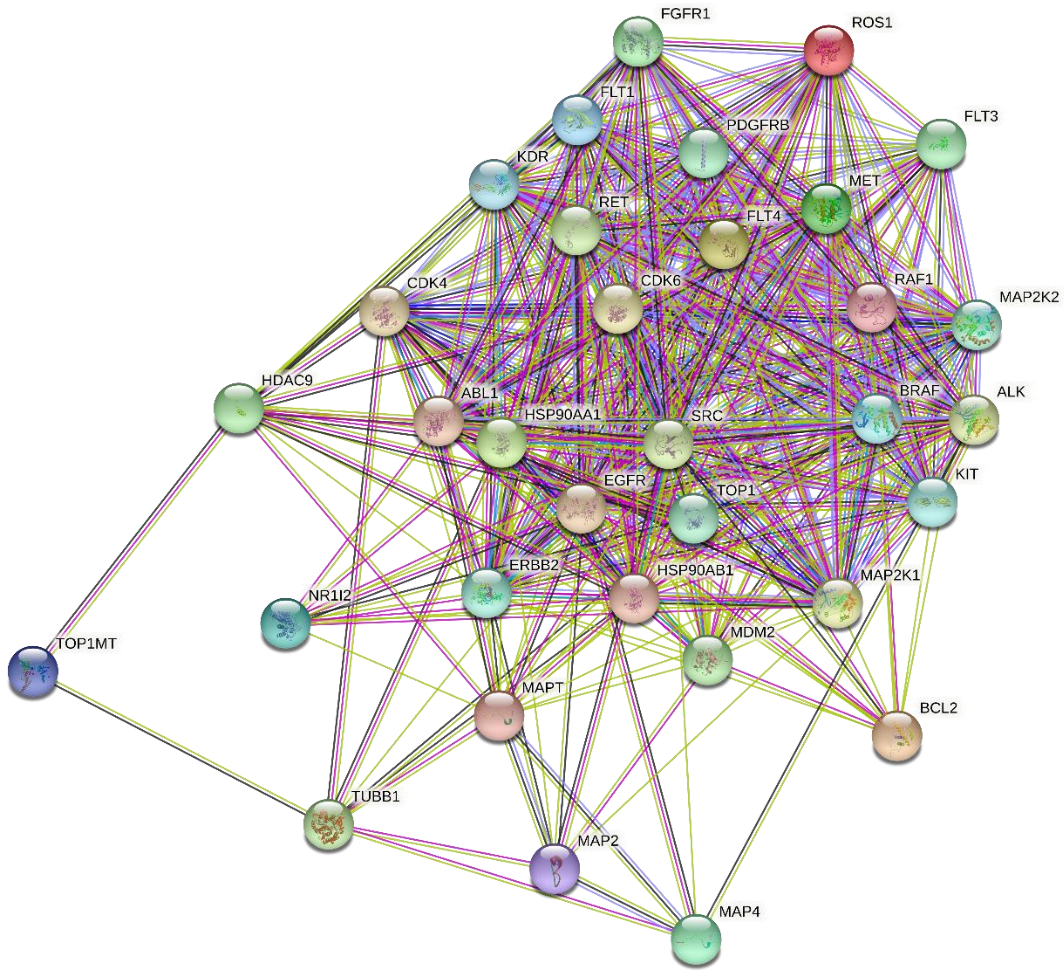

To compute the PPI-based similarity of drugs (SD_P), we constructed a bipartite graph for each pair of drugs. To explain clearly, suppose that TP_i and TP_j are the sets of target proteins for drugs d_i and d_j, respectively. Bipartite PPI graph $G_{i,j}^{PPI}$ is constructed such that the set of nodes in one part is TP_i, the set of nodes in another part is TP_j and the edges are defined based on STRING PPI network. For example, assume that $TP_i = \{P_{i1}, P_{i2}, P_{i3}\}$ are the target proteins of drug d_i while $TP_j = \{P_{j1}, P_{j2}, P_{j3}, P_{j4}\}$ are the target proteins of drug d_j. A schematic view of $G_{i,j}^{PPI}$ is illustrated in the following figure.

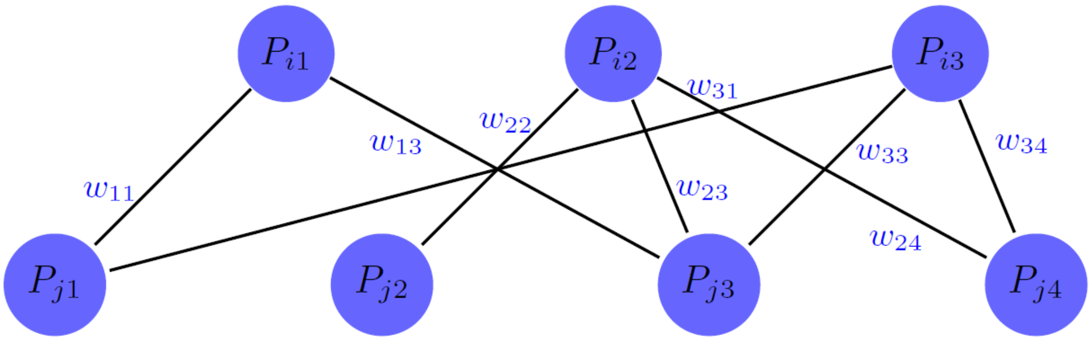

The weight of PPI edges between nodes of two parts in this graph are set by the weights of PPI in STRING network. Afterward, we applied the maximum weighted matching algorithm on $G_{i,j}^{PPI}$ and consider the summation of weights of matching edges as the PPI-based similarity of drug pairs ($SD_P(d_i, d_j)$). This similarity shows the extent of accordance between the set of target proteins of two drugs. Therefore $SD_P(d_i, d_j)$ is a high value, if the set of target proteins for d_i and d_j match highly.

Since the interactions in STiTCH and STRING databases were obtained based on various features, the newly introduced similarities gave us a comprehensive insights about the drugs from various aspects of view such as Gene ontology, pathway, text mining features, biological evidences, co-expressions, and reliable databases.

We computed the newly introduced drug similarities SD_N and SD_P for all drug pairs in GDSC and CCLE. Moreover, we applied CDSML using SD_N or SD_P as the drug similarity matrix. The following table represents the CDSML performance using newly introduced drug similarities (with or without using cell line similarities) based on stratified 5-fold cross-validation.

DATASET	SC	SD	AUC	AUPR	ACCURACY	F1-SCORE	PRECISION	RECALL
**GDSC**	**Not used**	NET	0.9072	0.9354	0.83	0. 8637	0. 8392	0. 8899
**GDSC**	EXP	NET	**0. 9157**	**0. 9398**	**0. 8388**	**0. 8715**	0. 8422	**0. 9031**
**GDSC**	MUT	NET	0. 9147	0. 9373	0. 8350	0. 8652	0. 8388	0. 8949
**GDSC**	CNV	NET	**0. 9157**	**0. 9398**	0. 8387	0. 8714	**0. 8424**	0. 9028
**CCLE**	**Not used**	NET	0.957	0.9796	0.9012	0.9225	0.9431	0.9033
**CCLE**	EXP	NET	0.9447	0.9717	0.8911	0.9143	0.9316	0.898
**CCLE**	MUT	NET	**0.9605**	**0.9815**	**0.9063**	**0.9267**	**0.944**	**0.9106**

**CCLE**	CNV	NET	0.9447	0.9717	0.8913	0.9145	0.9316	0.8983

DATASET	SC	SD	AUC	AUPR	ACCURACY	F1–SCORE	PRECISION	RECALL
**GDSC**	**Not used**	PPI	0.9071	0.9353	0.8292	0.8638	0.8357	0.8943
**GDSC**	EXP	PPI	**0. 9157**	**0. 9373**	0. 8354	**0. 8714**	**0. 8423**	**0. 903**
**GDSC**	MUT	PPI	0. 9147	**0. 9373**	0. 8354	0. 8652	0. 8392	0. 8929
**GDSC**	CNV	PPI	0. 9147	0. 9372	**0. 8355**	0. 8653	0. 8390	0. 8934
**CCLE**	**Not used**	PPI	**0.9606**	**0.9815**	0.9063	0.9267	**0.944**	0.9106
**CCLE**	EXP	PPI	0.9448	0.9718	0.8916	0.9145	0.934	0.8961
**CCLE**	MUT	PPI	**0.9606**	**0.9815**	0.9063	0.9267	**0.944**	0.9106
**CCLE**	CNV	PPI	**0.9606**	**0.9815**	**0.9064**	**0.9269**	0.9437	**0.9111**

The computed criteria for CDSML using SD_N or SD_P in the above tables verifies the high performance of CDSML using these similarity matrices. Moreover, efficient CDSML performance when applying solely based on SD_N or SD_P and not using any kind of cell line similarities shows that the newly introduced similarity matrices can be used solely and without cell line similarities.

c.f. (reference in the revised materials)

– Revised manuscript: Section 2.2.3 on pages 5 to 7 as well as Table 2

–Supplementary Materials: Section 4 on page 8

7. Author need to merge 0.5.1.c and 0.5.2

Thanks for your consideration we merged these subsections in the revised manuscript

c.f. (reference in the revised materials)

– Revised manuscript: Section 2.3.1 on pages 8 and 9

8. Author should provide experimental justification for the threshold of 0.6 at line 177.

Thank you for pointing this out. The number 0.6 was the number of section in the previous version of the manuscript, in which we explained the procedure of finding the best threshold. The best threshold was determined based on the elbow-point in the Precision_Recall curve. The rationality behind selecting this threshold is the favorable balance between Precision and Recall at this point. We added this justification to the revised manuscript.

c.f. (reference in the revised materials)

– Revised manuscript: The last paragraph in Section 2.4 on page 10

9. In the case studies, literature evidence should be sought for the predicted drug-cell line associations, not just drug-tissue association in general.

We admit that providing specific literature evidence for drug-cell line association rather than drug-tissue association is more reliable and can validate the predicted labels with more certainty. Nonetheless, we cannot find more specific literature evidence for drug-cell line associations. GDSC drugs are not widely studied, and little information is available for them. Unfortunately, we cannot find more specific literature evidence.

10. More detail discussion should be made about the methodologies, results found and various assumptions.

We thank the reviewer for this helpful comment. We revised the manuscript and added more specific discussions to all sections. The detailed methodology and reasonability of the assumption are more are elaborated meticulously in the revised manuscript.

Additional clarifications

We tried our best to apply the reviewers' suggestions and handle the issues pointed out by reviewers.

We look forward to hearing from you in due time regarding our submission and to respond to any further questions and comments you may have.

Sincerely,

Fatemeh Ahmadi Moughari and Changiz Eslahchi
